# Institutionalizing documentation for WHO Nigeria country office visibility and improved donor relations, 2013–2016

**DOI:** 10.1186/s12889-018-6191-1

**Published:** 2018-12-13

**Authors:** Charity Warigon, Wondimagegnehu Alemu, Fiona Braka, Hallah Tashikalmah, Yared Yehushualet, Kulchumi Hammanyero, Samuel Bawa, David Oviaesu, Sisay Tegegne, Mustapha Umar Maiiyali, Anne Eudes Jean Baptiste, Peter Nsubuga, Collins Boakye Agyemang

**Affiliations:** 1 World Health Organization, Country Representative Office, Abuja, Nigeria; 2 World Health Organization, Regional Office for Africa, Brazzaville, Congo; 3 National Primary Health Care Development Agency (NPHCDA), Abuja, Nigeria; 4 Global Public Health Solutions, Atlanta, GA USA

**Keywords:** Institutionalizing documentation, WHO Nigeria country office visibility, Improved donor relations

## Abstract

**Background:**

The mandate and unique experience of the World Health Organization (WHO) globally, enables over 190 countries, Nigeria inclusive, to depend on the technical support provided by the organization to define and mitigate the threats to public health. With other emerging health actors competing for scarce donors’ resources, the demand for visibility has invariably equaled expectations on WHO’s expertise and technical support. However, the inability to systematically document activities conducted by WHO personnel before 2013 overshadowed most of its invaluable contributions due to poor publicity. The inauguration of the Communications Group in December 2013 with a visibility plan necessitated a paradigm shift towards building a culture of documentation to engender visibility.

**Methods:**

We used a pre-post design of activities to evaluate the effectiveness of specific interventions implemented to improve visibility from 2013 to 2016. The paper highlights how incorporating communication strategies into the accountability framework of staff contributed in changing the landscape as well as showcasing the activities of WHO in Nigeria for improved donor relations.

**Results:**

With the specific interventions implemented to improve WHO’s visibility in Nigeria, we found that donor relations improved between 2013 and 2015. It is not a mere coincidence that the period corresponds with the era of incorporation of documentation into the accountability framework of technical staff for visibility as locally mobilized resources increased to record 112% in 2013 and 2014. The intervention assisted in the positive projection of WHO and its donors by the Nigeria media.

**Conclusion:**

Despite several interventions, which worked, made WHO ubiquitous and added awareness and visibility for donors who funded various projects, other factors could have contributed towards achieving results. Notwithstanding, incorporating documentation component into the accountability framework of field staff and clusters has significantly improved communication of WHO’s work and promoted healthy competition for increased visibility.

## Background

The mandate and unique experience of the World Health Organization (WHO) globally, enables 190 countries, Nigeria inclusive, to depend on the technical support provided by the organization to define and mitigate threats to public health [1, 2]. In a competitive environment, with emerging health sector actors competing for scarce donors’ resources the demand for visibility equals expectations on expertise and technical support. Accordingly, regular publications and sustained visibility of the activities and work of WHO in Nigeria is necessary and imperative [3, 4].

For the WHO Nigeria Country Office (WCO), the timely support provided to national authorities to achieve major milestones in disease surveillance and response is mainly due to the availability of competent personnel in the states. However, before 2013, the inability to systematically document activities of WHO personnel undermined the visibility of most of its contributions resulting in irregular information to update donors on activities implemented with their resources in Nigeria.

By the end of 2012–2013 Biennium (which is a standard WHO operational system implemented over 2 years), the WCO in Nigeria had the largest workforce in the African WHO Regional Office (AFRO) with 2605 personnel. Equally, WCO Nigeria had accounted for > 20% of the total AFRO budget with funding of > $93,700,000, provided in part by AFRO Member-States and other donors.

Concerned by the low visibility of its activities, the WCO in Nigeria in December 2013, constituted a Communications Group to improve its visibility. Several interventions were adopted to document and showcase the activities conducted across the six programmatic areas in the states and the Federal Capital Territory or FCT (e.g. Administration; HIV/AIDS, Tuberculosis and Malaria; Disease Prevention and Control; Family and Reproductive Health; Health Systems Strengthening and Immunization, Vaccines, and Emergencies).

The process instituted documentation of innovative strategies as well as the implementation of special interventions into the accountability framework of Zonal and State coordinators for improved visibility and increased donor relations as part of the communication strategy. Those interventions demonstrated WHO’s competence in the health sector, improved community health vis-à-vis overall donors and stakeholders contributions.

Previous studies by Haq Z et al. to document the implementation status of public-private mix (PPM) in 6 member countries of the WHO Eastern Mediterranean Region concluded that the implementation of country-specific communication plans to carry out local-level advocacy, strategic communication and effective social mobilization maximize the benefits of PPM [5]. Another example is the United Nation’s (UN) Central Emergency Response Fund (CERF) secretariat resource mobilization, communications and advocacy efforts [6]. These studies illustrate that strategic communication justified the need to institutionalize documentation for increased visibility and improved donor relations in public health interventions. Existing literature strengthens the role of communication in disseminating information on the leadership of WHO in coordinating the response to public health events [7].

Thus, this paper highlights the roles and contributions of WHO Nigeria personnel in changing the landscape and increasing the visibility of the activities implemented by WHO in Nigeria for improved donor relations.

## Methods

We used a pre-post design of activities to improve visibility from 2013 to 2016 to evaluate the impact of communication interventions carried out within the period under review. The evaluation focused on systematic increase on weekly contributions by zones and clusters which kept the website regularly updated with high-profile activities as they occurred from the partnership between WHO and the Ministry of Health and other partners to improve the health of Nigerians.

The study also assessed the donor resources mobilized during the period under review to test the relationship between increased visibility and total funding accruable to WHO.

### Structure of the WCO Nigeria and setting up WCO communication group

The WCO works towards the attainment of the highest level of health by all people in Nigeria through collaboration with the government and other partners in health development**.** The head office is in Abuja, with six coordinating zonal and 37 field offices in the 36 states of the country and the FCT. These offices provide technical support to the states and local government areas (LGAs). Each Zone has a coordinator, and each state is supervised by a Coordinator. This network office, in collaboration with partners and stakeholders enables the Organization to provide timely responses to health needs across the Federation.

The WCO Communication Group was inaugurated in December 2013. Shortly after, the Group presented a visibility plan towards the building of a culture of documentation to engender visibility. The plan was developed based on a situation analysis and a survey that included SWOT (Strengths, Weaknesses Opportunities, Threats) analysis and interviews with key officers in WHO, the UN system and the media. The objective was to triangulate information on the prevailing understanding or perception of WHO’s work in Nigeria by the WCO staff, other partner UN agencies and the public vis-a-vis WHO’s mandate. Additionally, the Country Cooperation Strategy (CCS), was analyzed to understand how its work aligned with WHO’s mandate in Nigeria.

A key outcome was a communication strategy, which increased the visibility of WHO’s work. Responsible persons for the timely implementation of activities were assigned to different roles and responsibilities. The strategic communication plan was first given to WCO staff at the end of 2013 staff annual retreat in Abuja. Subsequently, technical support for capacity building and orientation on how to document and showcase special interventions by WHO personnel across different programmes was given to field staff during quarterly review meetings.

### Incorporation of the accountability framework

From then onwards, the WHO Country Representative (WR), second level supervisors in the Zones and State Coordinators mainstreamed documentation in their activities and inculcated the culture of documentation [8]. The WR shared the status of states’ contributions to both Zonal and State Coordinators with written and verbal communication given for either recognition or sanctioning. Furthermore, at every quarterly meeting, a plan for documentation of at least two website uploads and visibility materials around thematic areas germane to WHO mandate was developed for enhanced monitoring implementation.

### Monitoring accountability

The Communication Group uploaded -articles on activities implemented by states with donor funding and in partnership with other stakeholders. The WCO provided quarterly feedback on the status of states’ contributions as uploaded on the website. The feedback was critical in completing the WHO staff Performance Management Development System (PMDS) as well as documenting polio legacy in the country. The introduction of the accountability framework undoubtedly contributed to improving staff performance and program deliverability consistently. It motivated those performing well, identified those performing below expectation and offered appropriate supportive and administrative actions. As a consequence, the accountability framework increased donors and partners confidence on WHO’s stewardship. This new level of relationship elevated WCO to continue disseminating results to showcase value for money to donors and intensified local resource mobilization efforts.

### Interventions

Website uploads: ---With weekly updates by zones and clusters, the website was regularly updated. That reflected International Health Days and high-profile activities as they occurred from the partnership between WHO and the Ministry of Health and other partners to improve the health of Nigerians. Those activities were geared toward responding to outbreaks and blocking cross-border disease transmissions.

A. Brochures:

The WHO bi-annual brochure was produced with articles already uploaded on the website. Such articles which might not have been in the public domain were printed for durability. The brochure highlighted contexts, interviews with stakeholders and presented human-interest stories from community beneficiaries. Each edition was sent to the 36 Commissioners for Health, 774 LGA chairpersons, 774 Directors of Primary Healthcare as well as prominent traditional and religious leaders. It was generally used to disseminate advocacy materials to mobilize additional resources among the stakeholders. Two editions were produced each year.

B. Peer-reviewed articles:

One of the key functions of the communications strategy is the publication and dissemination of operational research findings, best practices and special interventions to partners, donors, and government agencies. A supplement with 15 articles on some public health programmatic innovations implemented by the Nigerian government and its polio partners, with the support of the WHO to interrupt the transmission of polio virus was published in the *Journal of Infectious Diseases* for the first time in Nigeria [9]. Other reputable publications also published articles on the response to the Ebola disease virus, Lassa fever and f the HIV/AIDs response [10, 11].

C. Media materials and coverage:

Press releases were produced and distributed. WHO liaised with international, national and sub-regional media organizations to issue press releases at the beginning of an action or to provide detailed reports on important activities that the media might have missed. The media organizations also covered and reported on key WHO events such as the launching of the Country Cooperation Strategy, presentation ceremonies and commemoration of International Health Days.

D. Distribution of branded visibility materials:

Branded materials like annual reports, greeting cards, calendars, diaries, banners, caps, jackets were regularly distributed to government officials, traditional and religious leaders, donors, and partners and placed at public spaces. In addition, audio-visual materials such as documentaries were produced and widely disseminated on Radio and TV stations during prime time broadcast hours.

E. Recognition:

Best performing states were identified and presented with plaques to promote healthy competition amongst the states and clusters. The commemorative award ceremony during the end of year staff retreat was conducted after reviewing the status of states ‘contribution and total uploads on the website or contributions of visibility materials by states in the zones.

### Evaluation

We evaluated the impact of institutionalizing documentation by analyzing the status of states’ contribution by quarter. Since the website is WHO’s window to the world, we conducted monthly page views and users of the WHO Nigeria Country Office website.

We also assessed the donor resources mobilized during the period under review to test whether there was a relationship between increased visibility and total funding accruable to WHO.

## Results

**Fig. 1:**
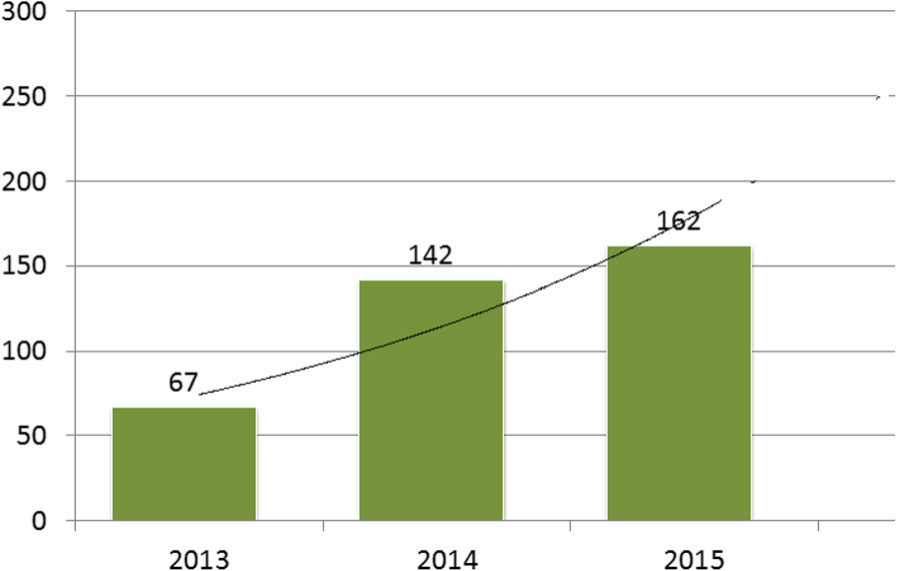
Increment in total donor resources mobilized by WHO Nigeria Country Office 2013–2015

Comparing January and February of 2013 with the corresponding period in 2014, did not indicate much difference in the number of page views or “visits” to the website. Figure 1 shows that changes began to occur from March of 2014 when 45, 876 users accessed the website. Additionally, page views increased from 127,243 to 173,119 in March 2014. From January to December, 2014, analysis of WHO’s website showed a rise in the number of “visits or page views” to 3,226,304 with 1,360,497 users representing an increase of 237% in new users or visitors. The numbers contrasted with 1,261,131 page views or visits and 512,759 users for the corresponding period in 2013. The period, July–October, 2014 especially showed uncommon activity; this corresponded with the Ebola virus disease outbreak. The articles posted from the index case to the technical support provided by the WCO further drew attention to the expertise available in WCO and increased website uploads. The scenario could be the pedestal on which explanation for increased donor and stakeholder relations gained grounds for the same period.

**Fig. 2:**
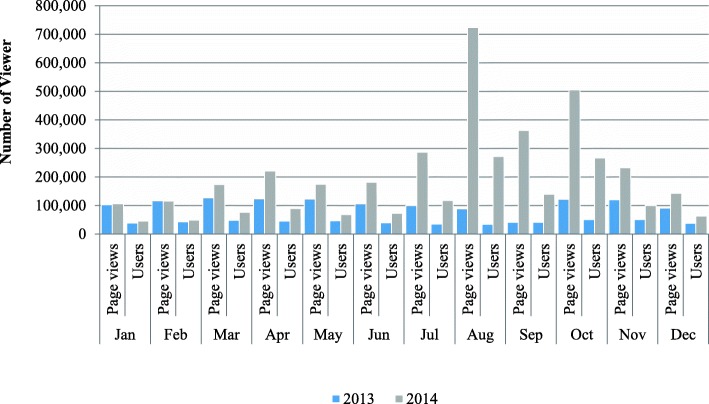
Number of Pageviews/users of WHO Nigeria website 2013–2014

Between January 2013 and December 2015, US$402 million was mobilized for both polio and non-polio programs. Figure 2 shows an increase of 112% in funding between 2013 and 2014 when donor contributions rose from $155 million to $506 million. In one single donation, the African Development Bank (AfDB) gave a grant of US $1 M to cushion the effect of the Ebola virus disease in Nigeria on 16th September 2014. The upward trend of resources mobilized continued through 2015 with more donor funding accounting for > 80% of total amount mobilized locally.

All the aforementioned documented activities that created visibility were implemented at no cost for WHO.

**Fig. 3:**
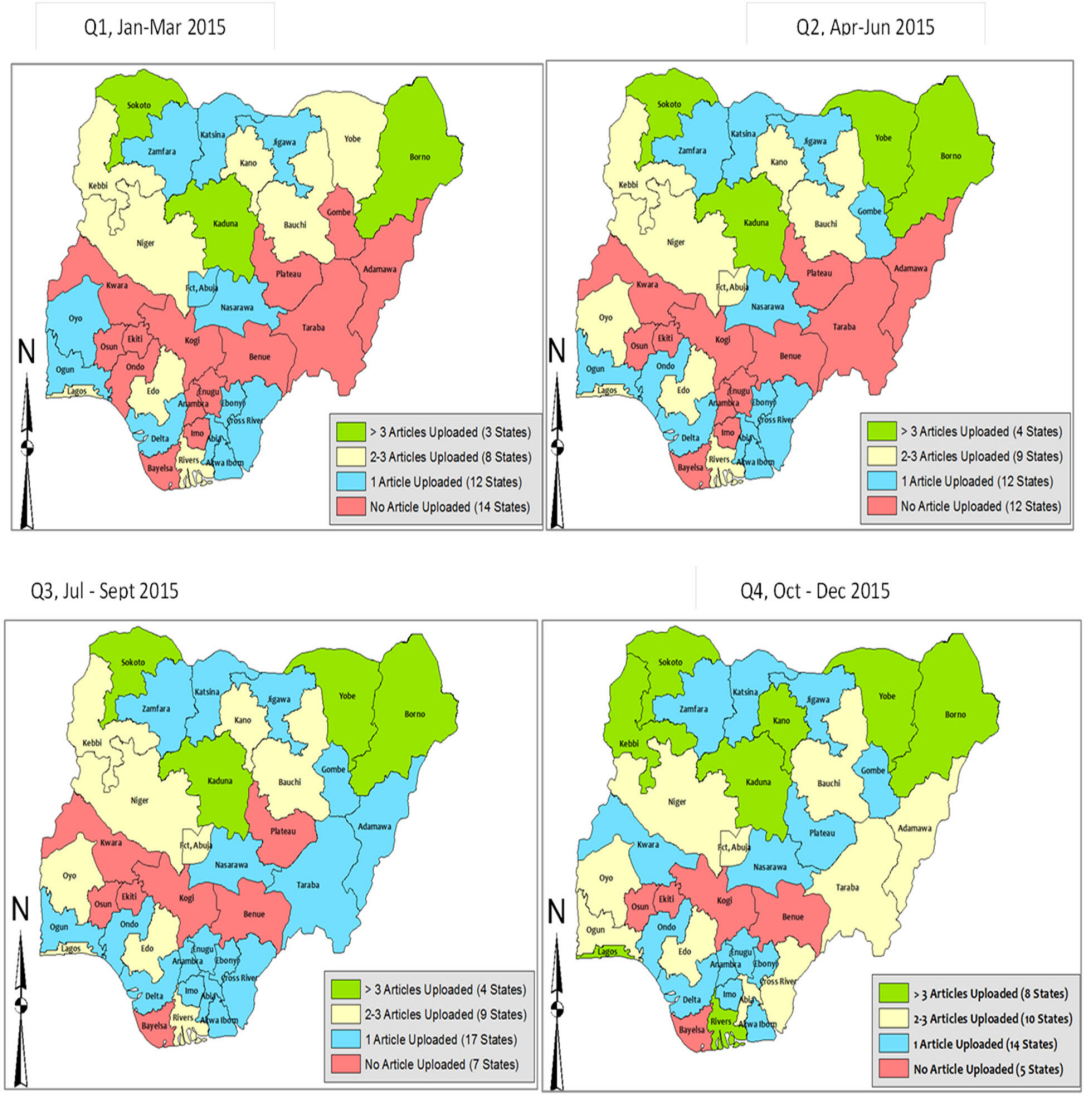
Status of articles uploaded by states on WHO Nigeria website state by quarter in 2015

Incorporating the visibility component into the accountability framework of Zonal and States’ coordinators resulted into an overall improvement in the documentation of WHO activities. Figure 3 shows that three states (Kaduna, Sokoto, and Borno) were consistent in documenting activities which were used as visibility materials beginning from the first quarter of 2014. From the second quarter of the same year, the frequency of uploads and updated overview of activities implemented by WHO state field offices increased. States in the South which hitherto made no contributions on the website uploaded articles thus increasing WHO’s visibility. By the third quarter of 2015, only Katsina and Kogi were yet to get any contributions uploaded on the website or used on other platforms for visibility.

Between December 2013 and 2015, > 20,000 copies of WHO branded materials that reflected the organization’s values were produced with dedicated names, WHO and donors logos for identity and visibility. During the same period, > 100 press releases were published and aired by the print and broadcast media on the works of WHO in Nigeria. Table 1 shows the various materials produced over a period of 3 years to change the narrative. For instance, three editions of WHO brochures were produced in 2014 unlike in the previous year when there was no documented material for reference to the work of WHO in Nigeria. These materials were shared with a wide range of stakeholders that constituted the target population across the 36 states and the FCT.

**Table 1:** Visibility materials on the work of WHO in Nigeria produced 2014–2016

Intervention	Target audience	2013`	2014	2015	2016
Website uploads/Press Releases	General public	7	48	66	75^a^
Peer-reviewed articles	Government agencies, donors, academics, researchers	3	3	5	21
Brochures	Donors, SMOH commissioners, traditional & religious leaders	0	2	6	3
Audio-Visuals	Donors, health workers	0	1	3	7
Cards/Calendars/diaries	General Public	0	1	6	4^a^
T-shirts/caps	General public				

^a^ Ongoing activity

Audio-visual materials, in a very important way, increased visibility for the work of WHO in Nigeria among public health workers and partners. Documentaries, short videos as well as radio and TV jingles were also produced to raise awareness on the reasons, results and impact of activities and projects implemented with donors’ resources.

## Discussion

In our review of the interventions, we found that increased documentation has led to increased WHO’s visibility in Nigeria. The increment upscaled donor relations, especially during the 2013–2015 period.. Consequently, timely and regular communications of articles produced by field staff built the confidence of donors and led to increased engagement with a resultant inflow of funds. In this regard, donors’ increased awareness of the utilization of their resources in project implementation tended to encourage them to increase their funding support.

In Nigeria, locally mobilized funds rose tenfold with cumulative funding requirements under WHO’s responsibility. That aligned with the perspectives of different schools that visibility is critical to improved donor relations. Indeed, some authors argue that the more people know about the accountable and positive utilization of their resources, the more inclined they are to be in providing additional support because they are guaranteed higher impact [12, 13].

Furthermore, our intervention revealed that with zero budgets for activities on increasing visibility, a non-governmental organization such as WHO can raise its profile and build a strong brand among stakeholders, donors and opinion leaders.

We have also observed that with regular updates of the website platform, the number of ‘visits, page views or hits’ to the website increased which boosted WHO’s presence on the internet; an easy public domain for donors. Therefore, it can be assumed that the rise in human interest articles contributed by field staff increased the frequency and appealed to divergent groups, with real life examples, from inside or outside of the organization. That way, people have tangible proof of the impact and benefits of the public health action that WHO recommended around a particular theme, goal or issue. The effect is that stakeholders have tended to feel more comfortable to rely on the site for information not only for issues at hand but also for the one after that.

We also found out that our interventions inculcated the culture of documentation as members of staff improved on their performances. More importantly, the incorporation of documentation into the accountability framework of Zonal and State coordinator instilled the culture of documentation in the system. There is, therefore, in effect a direct linkage with the WHO’s result based management approach that calls for the systematic implementation of the accountability framework by WHO country office in Nigeria, and enhanced performance in documentation across the states and all areas of work. Our report observed an immediate upsurge in the total contributions of high-profile activities that boosted the profile of WHO in Nigeria.

In 2013 when there was no mechanism to monitor documentation of activities, only seven (7) articles were uploaded on the website, the primary platform for providing updated overview of the organizations. However, from 2014 to June 2016 there was a steady increase in the number of visits/hits to the WHO website. The position of this paper aligns with the position of Tegegne et al. (2016) that enforcing the accountability framework improves performance and, therefore, must have been a major driving factor in increased documentation [8, 14, 15].

We further found that with successful documentation process in place, personnel at all levels were motivated to share best practices in their outstanding performances through publication of peer-reviewed articles which drew attention to the works of the country office in Nigeria. Before the institutionalization of the documentation process, few articles were published on WHO Nigeria best practices to share lessons learnt that could be replicated for further public health security. This paradigm shift translated into improved strategic communication with 34 out of 36 States and the FCT contributing to WHO visibility activities. Having internalized the processes, personnel focused on achieving results.

By the end of 2014, WHO was packaging, producing and distributing various branded visibility materials including brochures, cards, calendars, T-shirts, caps and audio-visuals to target audiences that appeal to donors’ projects such as commissioners for health, local government chairpersons, traditional and religious leaders as well as health workers.

Despite our several interventions which worked, made WHO ubiquitous, and increased awareness and visibility for donors, this study has some limitations. First, quality-scoring instruments for contributions have a bias towards uploaded articles on the website. Systematic reviews of others that did not make it to the website was not included. Many articles in our sample aptly documented the work of WHO in Nigeria but not necessarily approved for the website. Therefore, this may not be the most appropriate yardstick to judge or measure the effectiveness of interventions in attracting donors’ attention to influence the decision to increase funding. The reasoning is even more real, with the existence of extraneous contributing factors. Secondly, we may have created visibility, but there is no feedback mechanism to monitor how information is processed or decoded by our numerous stakeholders, including donors for improved relationship.

## Conclusion

Notwithstanding the possible limitations, incorporating documentation component into the accountability framework of field staff and clusters has significantly improved communication of WHO’s work and promoted healthy competition for increased visibility. Also, it has assisted in the positive projection of WHO and its donors by the Nigerian media.

The WHO Africa Regional Office can engage an independent agency to assess the divergent opinions on documenting coordinated actions on the broad social determinants of health. Such an assessment can also examine the effect of WHO’s technical support and its impact on other strategies such as behaviour change communication, risk communication, and social marketing for improved visibility.

## Abbreviations

AfDB: African Development Bank
AFRO: World Health Organization Regional Office
CCS: Country Cooperation Strategy
CERF: Central Emergency Response Fund
FCT: Federal Capital Territory
IEC: Information Education and Communication
IPDs: Immunization Plus Days
LGAs: local government areas
NPEEP: Nigeria Polio Eradication Emergency Plan
OPV: Oral polio vaccine
PHC: Primary health care
PMDS: Performance Management Development System
PPM: public-private mix
SWOT: Strengths, Weaknesses Opportunities, Threats
UN: United Nation
WCO: World Health Organization Nigeria Country Office
WHO: World Health Organization
WR: World Health Organization Country Representative
